# Chicago Medical College

**Published:** 1874-03-15

**Authors:** 


### CHICAGO MEDICAL COLLEGE. — PUBLIC COMMENCEMENT EXERCISES.

THE Annual Commencement Exercises of this institution were held in the amphitheater of the College, on Tuesday afternoon, March 10th, 1874, commencing at 2 o'clock P.M.

The large room was closely crowded with an audience of gentlemen and ladies. C. H. Fowler, President of the Northwestern University, presided, and conferred the Degree of Doctor of Medicine on forty-four candidates, the *Ad Eundem* Degree on one, and the Honorary Degree on one. The names of the graduates are as follows:

ORDINARY DEGREES. — Mortimer David Allen, Washington Beale Anderson, William Clarence Bedford, James Charles Bigelow, Horace Henry Briggs, Henry James Brooks, Xenophen Chapman, Willis F. Cobb, Lewis Samuel Cole, Edward DeWitt Converse, Lucien Charles Cowles, James Bennet Corr, Marion Carroll Dale, Edmund James Downing, Noble Fillmore Felker, William Herron Gale, Henry Gradle, James Isaac Hale, Wilford F. Hall, William Hausman, William Gardiner Hill, Charles Hervey Hunt, George Merrit Illingworth, Alexander Porter Kell, Gideon P. Kidd, Vallorous Frank Kinney, Frederick Falkenberg Laws, James Martin McClanahan, Edson Carey Miller, John Hestor Mitchell, Wilmot Leland Ransom, Frank C. A. Richardson, Frederick Julius Schlieman, Elijah Jaffries Snitcher, Charles Chester Sperry, Henry Joseph Stalker, John Christian Sundburg, John David Tritton, William Foote Whyte, Edwin Percy B. Wilder, George Edwin Willard, George Washington Willeford, Frederick C. Winslow, Dallis M. Wick.

AD EUNDEM DEGREE.—Loyal Firman Crawford, M.D.

HONORARY DEGREE.—Charles C. Hamrick.

The President accompanied the conferring of the degress by a short, but highly appropriate and impressive, charge to the graduates, which was responded to, on behalf of the class, by Mr. Kinney, whose address, both in style and sentiment, was admirable.

The prize for the best thesis was awarded to Mr. H. Gradle; and that for the second best, to J. H. Mitchell.

The exercises were varied, at suitable intervals, by good music, and were closed by an excellent general valedictory address, by Prof. H. P. Merriman.

The spring and summer course of instruction in the College will commence on Monday, the sixth day of April next, and will be very profitable to all such students as can remain in the city.

